# Aminolysis-mediated single-step surface functionalization of poly (butyl cyanoacrylate) microbubbles for ultrasound molecular imaging

**DOI:** 10.1186/s12951-024-02806-9

**Published:** 2024-09-01

**Authors:** Junlin Chen, Bi Wang, Anshuman Dasgupta, Céline Porte, Lisa Eckardt, Jinwei Qi, Marek Weiler, Twan Lammers, Anne Rix, Yang Shi, Fabian Kiessling

**Affiliations:** Institute for Experimental Molecular Imaging, RWTH Aachen University, 52074 Aachen, Germany

**Keywords:** Molecular imaging, PBCA microbubbles, Surface modification, Ultrasound contrast agent, Integrin, Peptide conjugation

## Abstract

Molecular ultrasound imaging with actively targeted microbubbles (MB) proved promising in preclinical studies but its clinical translation is limited. To achieve this, it is essential that the actively targeted MB can be produced with high batch-to-batch reproducibility with a controllable and defined number of binding ligands on the surface. In this regard, poly (n-butyl cyanoacrylate) (PBCA)-based polymeric MB have been used for US molecular imaging, however, ligand coupling was mostly done via hydrolysis and carbodiimide chemistry, which is a multi-step procedure with poor reproducibility and low MB yield. Herein, we developed a single-step coupling procedure resulting in high MB yields with minimal batch-to-batch variation. Actively targeted PBCA-MB were generated using an aminolysis protocol, wherein amine-containing cRGD was added to the MB using lithium methoxide as a catalyst. We confirmed the successful conjugation of cRGD on the MB surface, while preserving their structure and acoustic signal. Compared to the conventional hydrolysis protocol, aminolysis resulted in higher MB yields and better reproducibility of coupling efficiency. Optical imaging revealed that under flow conditions, cRGD- and rhodamine-labelled MB, generated by aminolysis, specifically bind to tumor necrosis factor-alpha (TNF-α) activated endothelial cells in vitro. Furthermore, US molecular imaging demonstrated a markedly higher binding of the cRGD-MB than of control MB in TNF-α activated mouse aortas and 4T1 tumors in mice. Thus, using the aminolysis based conjugation approach, important refinements on the production of cRGD-MB could be achieved that will facilitate the production of clinical-scale formulations with excellent binding and ultrasound imaging performance.

**Graphical Abstract:**

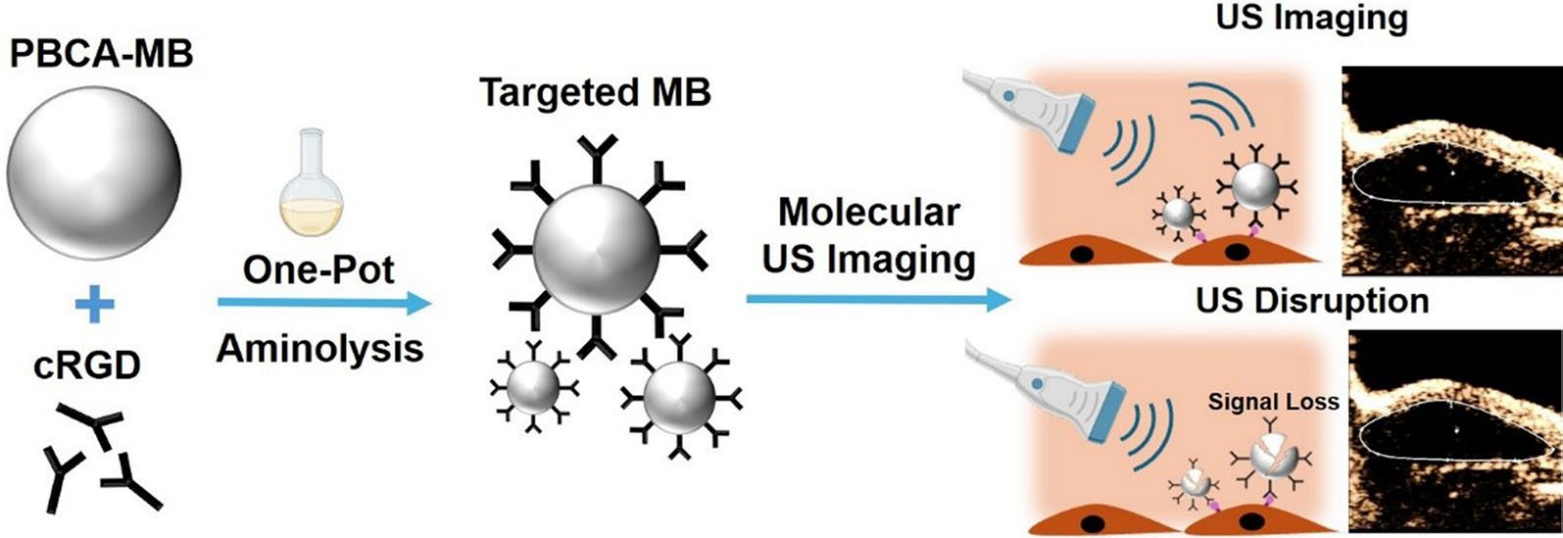

**Supplementary Information:**

The online version contains supplementary material available at 10.1186/s12951-024-02806-9.

## Introduction

Microbubbles (MB) are 1–10 micron-sized hollow air-filled particles whose shell is stabilized by lipids, proteins, or polymers [1]. They generate an ultrasound (US) signal due to the reflection of US waves at the interface of the gas core and the aqueous surrounding tissues [2, 3]. MB are typically used for intravascular US imaging, e.g. to visualize blood flow dynamics, as their large size confines the MB within the vasculature and restricts them from extravasating into tissues [4, 5].

For US molecular imaging, MB have been functionalized with targeting ligands such as antibodies and peptides to facilitate their binding specifically to the receptors overexpressed on the vascular endothelium [6]. Several targeted MB succeeded in preclinical studies, but only BR55 advanced to phase II trials [7]. To facilitate clinical translation, it is important that the production of actively targeted MB is easy, requires minimal steps, is batch-to-batch reproducible, acoustically stable, and preserves high ligand density and functionality. These criteria can be realized by choosing an appropriate shell composition, and by the coupling chemistry for the MB surface functionalization with ligands [8].

Despite being somewhat less echogenic than lipid-based MB, polymeric MB are particularly suited for US molecular imaging as versatile functionalization strategies enable simple and unlaborious coupling of targeting ligands. Among MB-stabilizing polymers, poly(butyl cyanoacrylate, PBCA) is a biodegradable polymer, well characterized, extensively used, and FDA-approved as a surgical adhesive for wound closing [9, 10].

PBCA-MB have been surface functionalized with antibodies and peptides using several strategies including non-covalent and covalent coupling methodologies [11]. Non-covalent coupling strategies are based on simple incorporation of a targeting ligand into the MB shell, such as physical absorption [12], electrostatic interaction [13] and the streptavidin–biotin technology [14, 15]. However, these methodologies have their own drawbacks. For instance, physical adsorption may result in premature release of targeting ligands, and streptavidin–biotin conjugation may lead to in vivo immunogenicity. Furthermore, covalent conjugation is more favorable as the formulations have better shelf-life stability. In this regard, we have shown that hydrolysis, followed by carbodiimide chemistry, can be employed to covalently couple targeting ligands on the surface of PBCA-MB [16]. However, this reaction is often difficult to control and leads to MB destruction [17]. Furthermore, these multi-step procedures are time-consuming, expensive, and it is often hard to reproduce and scale up these MB formulations. Thus, strategies are needed to covalently couple targeting ligands on PBCA-MB that can eventually be translated to the clinic.

Here, we developed a single-step coupling method based on aminolysis to bind targeting ligands, i.e. cRGD peptides, to the surface of PBCA-MB. cRGD peptides are known to bind strongly to α_v_β_3_ integrin, which are overexpressed in tumor vasculature. The covalently conjugated cRGD-MB showed high yield and low batch-to-batch variability. In comparison to untargeted MB, cRGD-MB were found to strongly bind to α_v_β_3_ integrin in both in vitro and ex vivo under flow conditions. Finally, US molecular imaging demonstrated that the cRGD-MB have markedly higher binding to the neovasculature of 4T1 tumor bearing mice. Overall, these findings lay the foundation for the clinical translation of actively targeted PBCA-MB formulations.

## Materials and methods

### Fabrication of PBCA-MB

PBCA-MB were synthesized based on anionic-emulsion polymerization as described previously [18]. Briefly, 3 mL of n-butyl cyanoacrylate (BCA, Special Polymers, Sofia, Bulgaria) was added drop-wise to 300 mL aqueous solution containing 1% of Triton-X100 at pH 2.5. This mixture was emulsified by Ultra-Turrax T-50 basic (IKA Werke, Staufen, Germany) at 10,000 RPM for 1 h at room temperature. The resulting solution was centrifuged at 500 RPM (46 G) for 10 min and washed with 0.02% (v/v %) aqueous solution of Triton-X100 (pH = 7, Sigma-Aldrich, Munich, Germany) until the subnatant was transparent. Size and concentration of MB were measured using a Coulter counter (Multisizer 4e, Beckman, Brea, United States). To perform Coulter counter measurements, a 5 μL solution of MB was mixed with 20 mL of ISOTON® II (Beckman Coulter, Brea, United States), and triplicate readings were taken.

### Functionalization of PBCA-MB

Cyclo(Arg-Gly-Asp-D-Phe-Lys) (cRGD, MedChemExpress, Monmouth Junction, USA) binds to α_v_β_3_ integrin on the cell surface with a pretty low IC_50_ of 0.94 nM [19]. To achieve effective binding of cRGD-MB to α_v_β_3_ integrin, cRGD peptides were conjugated covalently to the surface of the MB by direct coupling of the free amine group on the peptides to aminolysed ester bond of BCA backbone on the surface of the MB. To this end, 2.2 mg cRGD peptide was dissolved in 300 μL 20 mM 4-(2-hydroxyethyl)-1-piperazineethanesulfonic acid (HEPES, Sigma-Aldrich, Munich, Germany) buffer and subsequently incubated with 10^9^ PBCA-MB in HEPES (1 M)-Triton X-100 (0.01%) buffer (pH 7). Subsequently, 17 µL (0.05 mmol) of 0.01% Lithium Methoxide (Sigma Aldrich, Darmstadt, Germany) were added and the pH value was adjusted to 8 by adding 0.1 M NaOH (Carl Roth, Karlsruhe, Germany). The reaction was kept at room temperature for 24 h under continuous stirring at 300 RPM and then purified with HEPES/Triton X-100 buffer through centrifugation and washing steps. Cyclo(Arg-Ala-Asp-D-Phe-Lys) (cRAD, MedChemExpress, Monmouth Junction, USA) modified MB that do not bind to α_v_β_3_ integrin, were prepared identically and used as a negative control.

### High-performance liquid chromatography

The cRGD-MB solution was purified using a dialysis membrane (6-8 k MWCO, Spectrum, Massachusetts, USA) in ultrapure water to remove Triton X-100 and uncoupled peptides. The purified samples were then mixed with an equal volume of acetonitrile (ACN) to disrupt the MB to polymers prior to the characterization with high-performance liquid chromatography (HPLC). HPLC was performed using an Agilent 1260 Infinity system (Agilent technologies, Waldbronn, Germany) equipped with a diode array and multiple wavelength UV–Vis detector and a reversed phase column (Eclipse Plus C18, 3.5 μm, 4.6*150 mm). A gradient elution method was applied with eluent A (0.1% trifluoroacetic acid in ACN) increasing from 5 to 95% in 10 min (eluent B was H_2_O with 0.1% trifluoroacetic acid). The flow rate was set at 1 mL/min, and the injection volume was 50 µL. The UV–Vis detector operated at a wavelength of 220 nm to detect cRGD. Chromatograms were recorded and analyzed using the Agilent ChemStation software (Agilent technologies, Waldbronn, Germany).

### Gel permeation chromatography

To track the aminolysis reaction, PBCA-MB were coupled with fluorescent Cy3-amine (Lumiprobe, Hannover, Germany) instead of cRGD. The Cy3-coupled MB and unmodified MB were lyophilized and further dissolved in dimethylformamide (DMF) containing 10 mM LiCl at a concentration of 5 mg/mL. The resulting solution was filtered through a 0.22 µm membrane to remove any precipitate. Gel permeation chromatography (GPC) was conducted using a PLgel 3 μm MIXED-E column (300 × 7.5 mm, Agilent technologies, Waldbronn, Germany) and PEG-standards (Agilent technologies, Waldbronn, Germany) were applied as calibration standards according to the manufacturer’s instruction. The retention time of each compound was recorded with infrared (IR) and UV–Vis detector (550 nm wavelength for Cy3 detection) at the same time. The eluent was DMF containing 10 mM LiCl, the elution rate was 0.5 mL/min, and the temperature was 55 ℃. Data analysis was carried out using a specialized Cirrus GPC software provided by the instrument manufacturer (Agilent, Waldbronn, Germany).

### Cryogenic scanning *electron* microscopy

The morphology of standard-MB and cRGD-MB was investigated by cryogenic scanning electron microscopy (Cyro-SEM) with a Hitachi FE-SEM 4800 (Krefeld, Germany) equipped with an Alto 2500 Cryo-Gatan unit (Gatan GmbH, Munich, Germany) operating at 1 kV and 2 μA. Standard-MB and cRGD-MB were dropped onto a sample holder and frozen in liquid nitrogen. Subsequently, the sample holder was inserted into the preparation chamber for morphology observation. The MB droplets were fractured using a carbide knife and then sublimed at − 100 °C for 5 min to observe the structure and morphology of standard-MB and cRGD-MB.

### Flow cytometry

Cy3 was used as a model dye and was conjugated to the PBCA-MB both using the aminolysis and the hydrolysis protocol described in previous studies [18, 20] and investigated by flow cytometry (Becton Dickinson (BD) FACS Canto II, Heidelberg, Germany). The mean fluorescence intensity (MFI) of Cy3 per MB was used to quantify the level of conjugation of the ligand. As the aminolysis protocol may result in a different MB size distribution, the variability of the MB surface coverage with dyes was investigated by correlating MB sizes with MFI.

The detailed procedure was as follows: Purified Cy3-MB (5 × 10^6^/mL in a 0.02% triton solution) were measured at a low flow rate, and 50,000 events were recorded for each sample. Standard-MB were used as a negative control. The MFI was analyzed using flowjo-v10 (FlowJo LLC, Ashland, United States), in which standard-MB were gated as Cy3 negative.

### In vitro US phantom imaging

Custom-made gelatin-based phantoms were used to analyze the echogenicity of standard-MB and cRGD-MB. The VEVO 3100 US system, incorporating a linear MX-250 transducer (FUJIFILM VisualSonics, Toronto, Ontario, Canada), was employed for US imaging. 3 × 10^5^ MB were suspended in 4.5 mL of 2% w/v gelatin solution, and the mixture was embedded in 10% w/v gelatin solution. The transducer was fixed vertically above the phantom using a focus depth of 11 mm. US imaging was performed in non-linear contrast mode (NLC-mode) at 18 MHz frequency and 4% power. 100 frames were recorded, followed by a destructive pulse (0.5 s at 100% power) to destroy the MB in the gelatin phantom. To quantify the acoustic intensity, a region of interest (ROI) was drawn within sample-loaded gelatin and acoustic intensities were assessed before and after the US destructive pulse using the VevoLAB software version 3.2 (FUJIFILM VisualSonics, Toronto, Ontario, Canada).

### Cell culture

The binding efficiency of cRGD-MB to α_v_β_3_ integrin was investigated via Human umbilical vein endothelial cells (HUVEC). HUVEC were obtained from PromoCell (Heidelberg, Germany), maintained in Vasculife Basal Medium (Lifeline, Troisdorf, Germany) enriched with fetal bovine serum (FBS: 2% v/v), endothelial cell growth supplement, gentamycin (1% v/v), and 1% penicillin/streptomycin. For the in vitro flow chamber assay, HUVEC were grown in 1% gelatin pre-coated 35-mm petri dishes until they reached 80% confluency. 4T1 cells (murine epithelial mammary carcinoma) were used for the establishment of mouse breast tumor model. They were obtained from the American Type Culture Collection (ATCC, Manassas, Virginia, USA), and maintained in Roswell Park Memorial Institute Medium (RPMI 1640) with 10% fetal calf serum and 1% penicillin/streptomycin. Cells were passaged every three days.

### In vitro flow-chamber binding studies

The specific-binding studies of cRGD-MB, cRAD-MB and standard-MB were performed in an in vitro flow chamber model as described previously [21]. Briefly, 100 μL containing 10^6^ HUVEC/mL were added to 1% gelatin pre-coated 35-mm petri dishes. 4 h before the MB solution was added to the chamber, HUVEC were activated with 4 ng/mL of recombinant human TNF-α. The cells were then stained with Alexa Fluor 488Y-conjugated wheat germ agglutinin (WGA-AF488; Thermofisher, MA, USA) and nuclei were stained with Hoechst (Invitrogen, CA, USA) for 30 min, followed by washing 3 times with HUVEC medium. Petri dishes were placed in a flow chamber and a flow rate of 0.25 mL/min was applied using a peristaltic pump (Gilson Inc, Villiers-le-Bel, France). All MB were pre-dyed with rhodamine B (1 mg in 10 mL MB solution; Merck, Darmstadt, Germany) as described previously and were further purified by washing with HEPES/Triton X-100 buffer [9]. Subsequently, rhodamine-MB were added to the perfusion system at a concentration of 1 × 10^8^ MB /mL and allowed to circulate for 10 min in a closed loop. After the circulation phase, the petri dishes were disconnected from the flow chambers and washed with HUVEC medium to remove unbound MB. Fluorescence microscopy (Carl Zeiss AG, Oberkochen, Germany) was used to analyze the specific binding of MB to HUVEC.

### Ex vivo flow-chamber binding studies

Eight-week-old C57BL/6 J wild-type mice were used (n = 10). Mice were euthanized by cervical dislocation under anesthesia with 1.5% isoflurane (FORENE, AbbVieAG, Ludwigshafen, Germany) and subsequently their aortas were dissected. A custom-built flow chamber setup equipped with a pipette (tip diameter: 120–150 μm) was employed and the sink was filled with Ringer solution (B. Braun, Melsungen, Germany). The aortas were mounted end-to-end and the endothelium was activated by perfusion with 4 ng/mL TNF-α in Ringer solution for 4 h. The standard-MB, cRAD-MB, and cRGD-MB were introduced at a concentration of 10^8^ MB/mL and at a flow rate of 0.25 mL/min. To image the vessel aortas in high resolution, US measurements were performed with an MX-700 US transducer (Vevo 3100, FUJIFILM VisualSonics, Amsterdam, Netherlands) at a frequency of 50 MHz, a transmit power of 1%, and a dynamic range of 60 dB. After 10 min of circulation, the MB were removed by a perfusion with Ringer solution. Bound MB were detected using the US destruction-replenishment analysis method [22]: MB were disrupted by applying 100% US power in Doppler mode at a frequency of 40 MHz with a pulse repetition frequency of 5 kHz, duration of 10 s and dynamic range of 40 dB. 100 frames were recorded in B-mode before and after the destructive US pulse and analyzed using the software MATLAB (R2022a, Natick, Massachusetts, USA). The mean ultrasound intesity per frame was determined within a ROI that covered the vessel wall. Utrasound intensity-time-curves were plotted.

### In vivo molecular US imaging

All animal experiments were approved by the German State Office for Nature, Environment and Consumer Protection (LANUV) North Rhine-Westphalia. 10–12 week old female Balb/c mice (Janvier Labs, Le Genest-Saint-Isle, France) were housed on spruce granulate bedding (Lignocel, JRS, Germany) in groups of 3–5 animals in type II long individual ventilated cages (Tecniplast, Germany) under specific pathogen-free conditions. Husbandry rooms were temperature (20–24 °C) and humidity (45–65%) controlled. Water and standard pellets for laboratory mice (Sniff GmbH, Soest, Germany) were offered ad libitum. Group-housed animals were assigned individual earmarks for identification. Tumors were induced by subcutaneous injection of 4 × 10^4^ 4T1 cells in 50 μL phosphate-buffered saline (PBS) in the right hind limb. Tumor diameters were monitored by caliper measurements. After tumors had reached 5–6 mm in diameter, molecular US imaging was performed using the VEVO 3100 US system equipped with a linear MX-250 transducer. The transducer was positioned vertically over the tumor region and the focus depth was kept at the middle of the tumor. During all US measurements, mice were kept under inhalational anesthesia using 1.5% isoflurane. Imaging was performed in NLC-mode at 18 MHz and with 10% transmit power. Mice in groups of standard MB (n = 5), cRAD-MB (n = 5) and cRGD-MB (n = 5) were injected with 5 × 10^7^ MB in 50 μL 0.9% saline solution as a bolus via a tail-vein catheter, followed by a 20 μL saline flush. During the intravenous injection of the MB solution, the signal enhancement was assessed by recording 500 frames in NLC-mode (frame rate: 10 fps). The maximum inflow of MB was described by the peak enhancement. The intravenous injection of MB was followed by an interval of 8 min, during which the MB had time to bind to their target and unbound MB were cleared from the bloodstream. Thereupon, an NLC-mode image sequence was acquired where 100 frames were recorded before and after a 1-s-long destructive pulse at 100% power. Finally, the signal deriving from bound MB was quantified by subtracting the post- from pre-destructive US mean acoustic intensity using the VevoLAB software version 3.2 (FUJIFILM VisualSonics, Amsterdam, Netherlands).

### Histological analysis

After US imaging, mice were injected with fluorescein (FITC)-conjugated lectins (Vector Laboratories Inc, Burlingame, California, USA) and euthanized by cervical dislocation after 15 min. Tumors were resected, embedded in Tissue-Tek (Sakura Finetek Europe, Alphen aan den Rijn, Netherlands), snap-frozen and stored at − 80 °C until cryosectioning. Immunofluorescence stainings were performed on 8 μm-thick cryosections. The tumor sections were fixed with 80% methanol for 5 min at 4 °C followed by the addition of acetone at − 20 °C for 2 min. After fixation, sections were washed 3 times with PBS and incubated overnight with a rabbit anti-α_v_β_3_ integrin antibody (1 µg/ µL; eBioscience, San Diego, California, USA) at a dilution of 1:100 at 4 °C. Thereafter, the sections were washed with PBS and stained using a donkey Cy3-labeled secondary anti-rabbit antibody at a dilution of 1:500 (7.5 µg/ µL; Dianova, Hamburg, Germany). The mean percentages of the α_v_β_3_ integrin-positive areas to the total area were calculated using Image J (Carl Zeiss AG, Oberkochen, Germany).

### Statistical analysis

Data are shown as mean ± standard deviation (SD). To calculate statistical differences between two groups, the unpaired t-test was used. One-way analysis of variance (ANOVA) with subsequent Tukey post-hoc testing was applied for comparing more than two groups. All statistical analyses were performed using GraphPad Prism 10 (San Diego, California, USA). A statistically significant difference was considered at *p < 0.05; **p < 0.01; ***p < 0.001, as indicated in the figure legends.

## Results

### Preparation and characterization of cRGD-MB

**Fig. 1:**
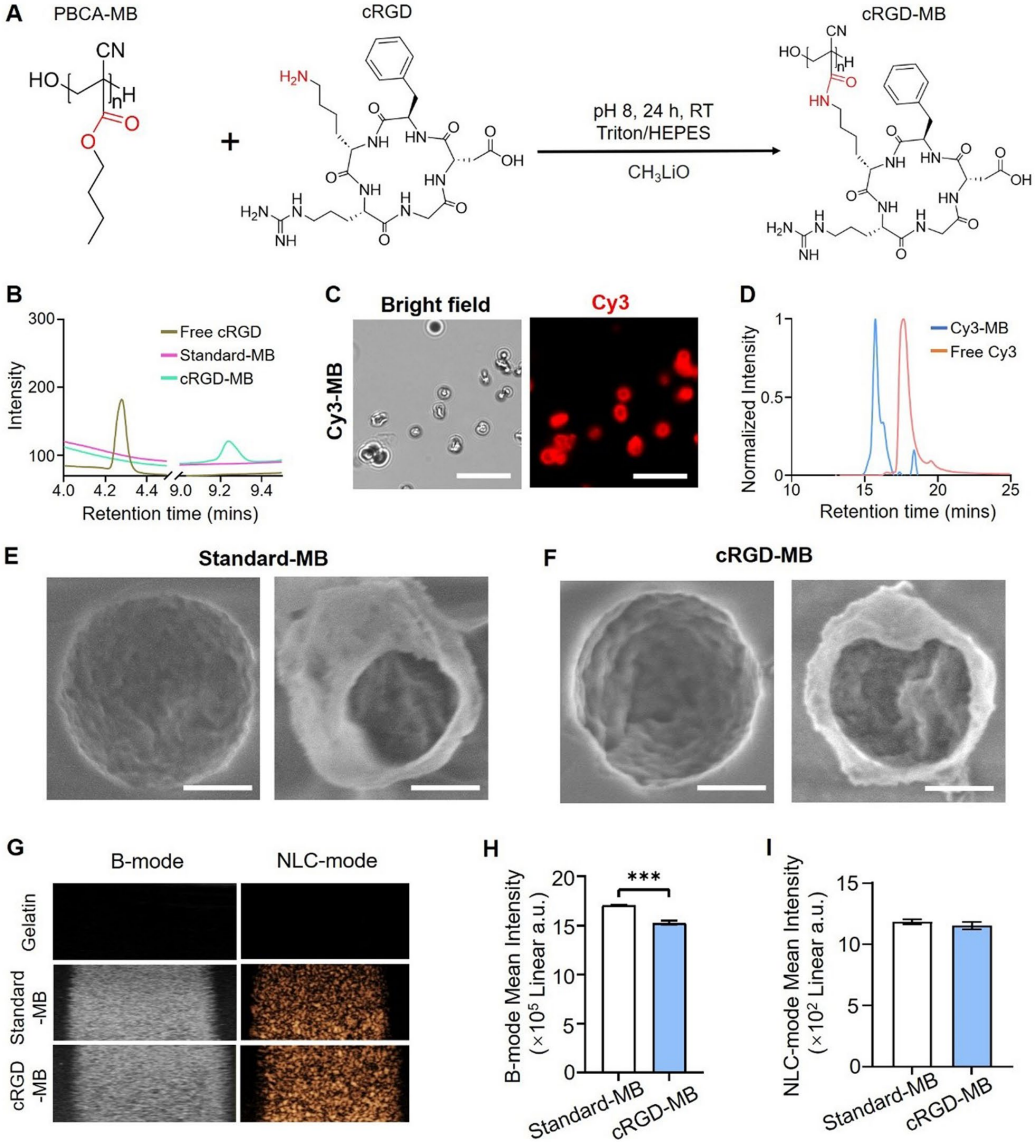
Preparation and characterization of cRGD-MB. **A** Scheme of the single-step functionalization using aminolysis on preformed PBCA-MB. **B** HPLC profile showing that the peak of cRGD-MB elutes later compared to free peptide cRGD, thus indicating the formation of a new compound. **C** Fluorescence images of MB after functionalization of Cy3-amine (Cy3-MB) using aminolysis. Cy3 signals were co-localized with the MB shell. Scale bar = 10 μm. **D** GPC analysis showed that the polymer of Cy3-MB had a distinctly shorter retention time than free Cy3-amine. **E**, **F** Cryo-SEM images of standard-MB (E) and cRGD-MB (F), respectively. The gas core–shell structure of MB was preserved after functionalization using cRGD. Scale bar = 500 nm. **G** Brightness mode (B-mode) and non-linear contrast mode (NLC-mode) images of 2% gelatin containing control, standard-MB, and cRGD-MB. The cRGD-MB generated by aminolysis were detectable in both US modes, indicating that the cRGD-modification via aminolysis did not change the US properties of cRGD-MB. **H, I** Quantification of the B-mode (H) and NLC-mode signal (I) of standard-MB and cRGD-MB. cRGD-MB were still detectable after functionalization. Data represent mean ± SD of three independent MB batches. Statistical comparisons were performed using unpaired t-test. ***p < 0.001

PBCA-MB were prepared by anionic polymerization of BCA. Using single-step aminolysis, actively targeted PBCA-MB were generated by adding cRGD peptides to preformed PBCA-MB with lithium methoxide as a base catalyst at pH 8 (Fig. 1A). To optimize the cRGD dosage in the aminolysis reaction, we employed a colorimetric peptide assay to evaluate ligand density on MB and immunoprecipitation to assess their targeting efficiency. The number of cRGD peptides per MB increased with the amount of cRGD peptide added during aminolysis (Fig. S1A). However, the MB targeting efficiency was saturated after surface modification density reached 2.31 × 10^–9^ μg/MB cRGD (Fig. S1B). Therefore, we selected 2.2 mg of cRGD as the optimized dosage for aminolysis.

Several characterization techniques were used to confirm the covalent conjugation of cRGD onto the surface of the PBCA-MB. HPLC chromatograms showed no peak for the polymers of standard-MB and a peak at low retention time for the cRGD peptides alone. In contrast, the polymers of cRGD-MB had a notably higher retention time (9.0 min vs. 4.3 min), thereby indicating cRGD coupling on the PBCA-MB (Fig. 1B). To further confirm the conjugation chemistry, PBCA-MB were also coupled with Cy3-amine instead of cRGD and characterized using fluorescence imaging. As expected, we clearly detected Cy3 signals on the MB shell (Fig. 1C). The Cy3-coupled MB (Cy3-MB) were further characterized by GPC, showing that the specific Cy3 absorbance of Cy3-MB was eluted earlier than free Cy3-amine (15.7 min vs. 17.7 min; Fig. 1D). These results show that the polymer population of Cy3-MB has higher molecular weight than free Cy3-amine, confirming the successful formation of Cy3-MB. Cy3-amine functionalized MB have a wide molecular weight distribution, characterized by a bimodal distribution as revealed by GPC (Fig. S2). The functionalized PBCA (Peak I) shows a Cy3 signal under the UV–Vis detector, which is not present in the unfunctionalized PBCA (Peak II), indicating that the functionalization is non-uniform. This is likely because the hydrophilic Cy3-amine and peptides can only be covalently bound to the outer part of the MB surface and cannot diffuse into the hydrophobic MB shell (the latter also not being intended).

Cryo-SEM and US were employed to study the morphology and acoustic properties of cRGD-MB. Cryo-SEM images show that both cRGD-MB and standard-MB were similar in structure and morphology (Fig. 1E–F). When exposed to US, both standard-MB and cRGD-MB exhibited visible B-mode and NLC-mode US signals (Fig. 1G–I). The mean B-mode signal intensity of cRGD-MB was only ~ 10% lower than that of standard-MB (Fig. 1H). Additionally, cRGD-MB synthesized via aminolysis remained stable in suspension without significant alteration of their size and concentration for over 7 days (Fig. S3). These findings indicate that the aminolysis procedure does not negatively impact the structure, morphology, and acoustic properties of cRGD-MB.

**Fig. 2:**
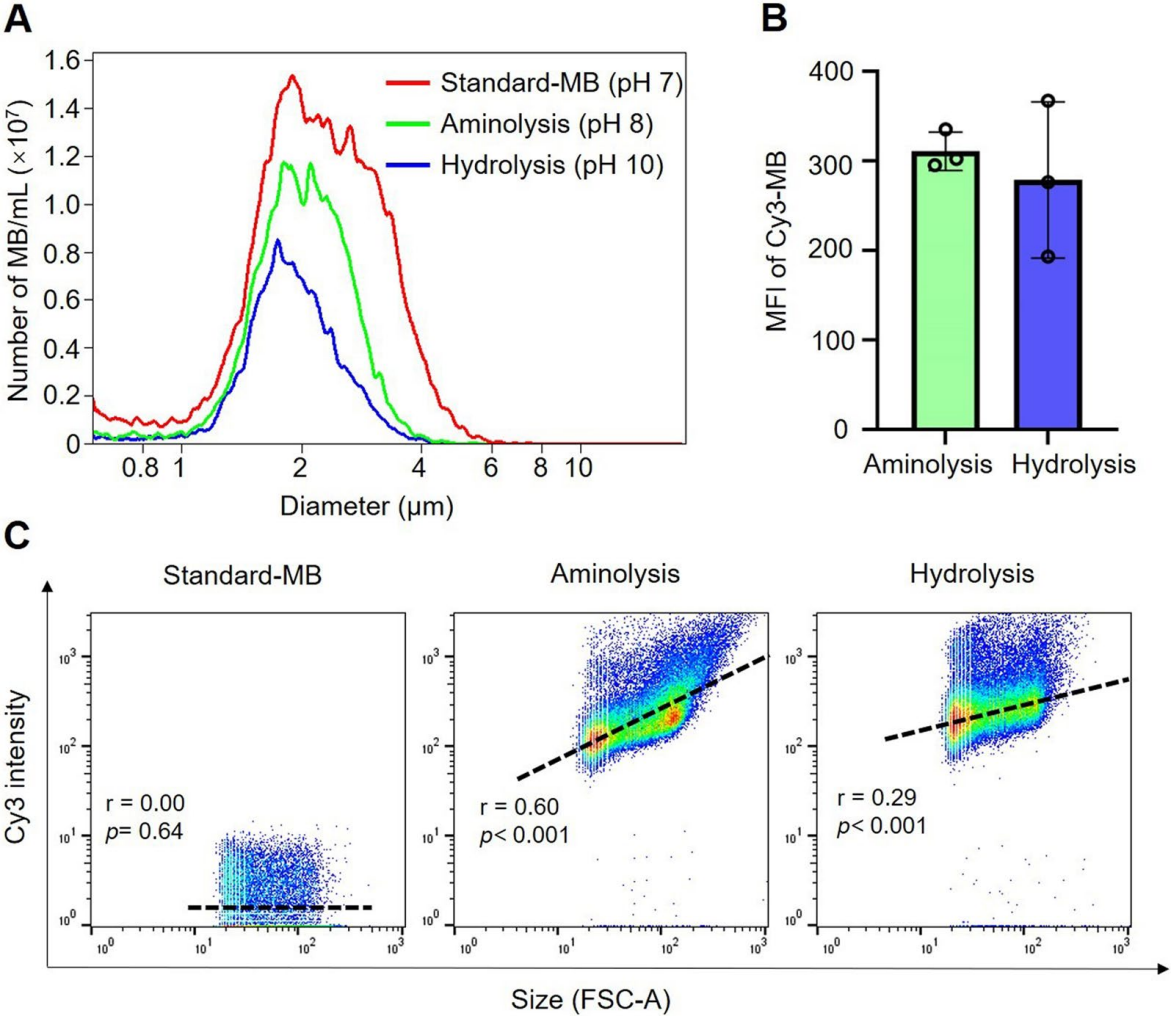
Comparison of MB yield and coupling efficiency using conventional hydrolysis vs. aminolysis.** A** Concentration and size distribution of uncoupled, standard-MB (red) as well as targeted cRGD-MB obtained through aminolysis (green) and hydrolysis (blue) detected by Coulter counter. The results indicate that the modification via hydrolysis significantly decreased the number of MB, while aminolysis at pH 8 largely protected MB from degradation. **B** Batch to batch reproducibility: The mean fluorescent intensity (MFI) of different batches of Cy3-MB prepared by hydrolysis or aminolysis analysed by flow cytometry. Both protocols resulted in comparable MFI, while Cy3-MB prepared by aminolysis showed lower variation in MFI. Data represent mean ± SD of three independent MB batches. Statistical comparisons were performed using an unpaired t-test. p ≥ 0.05. **C** Homogeneous dye coupling efficacy: FACS analysis showing the correlation between dyes on the MB surface and MB size. Via aminolysis, a more consistent number of bound dyes was achieved for MB of different sizes as compared to hydrolysis (indicated by the r-values). Statistical dispersion was analyzed by simple linear regression

We finally compared the efficiency of the aminolysis conjugation to a previously commonly used coupling strategy employed for PBCA-MB. There, PBCA-MB were hydrolyzed to allow for subsequent carbodiimide coupling of antibodies and peptides [16, 20, 23]. We found that higher Cy3-MB yields were obtained using aminolysis than hydrolysis (71.1 ± 11.9% vs. 43.6 ± 14.4% MB survived the conjugation chemistries; Fig. 2A). These findings can be attributed to the fact that hydrolysis at pH 10 often results in depolymerization [11], thereby causing MB destruction. In addition, aminolysis uses mild conditions, i.e. pH 8, and hence better maintains the integrity of the MB [24]. To compare the coupling efficiency, we used Cy3-amine as a model ligand and quantified its conjugation to MB by flow cytometry using both coupling methods. The aminolysis protocol resulted in comparable Cy3 binding efficacy (Fig. 2B) but better controlled surface coverage (higher correlation of MB size and bound dyes: for aminolysis r = 0.60 vs. hydrolysis: r = 0.29; Fig. 2C) and improved batch to batch reproducibility (mean fluorescence intensity: 310.7 ± 21.36 vs. 278.7 ± 87.03; see lower standard deviation in Fig. 2B). We further investigated the amount of conjugated cRGD per MB by performing a colorimetric peptide assay. Consistent with the results of flow cytometry, lower batch-to-batch variation was observed in the aminolysis approach (2.2 ± 0.4 × 10^–9^ μg / MB, equal to around 2.19 × 10^6^/MB) compared to hydrolysis (1.7 ± 1.3 × 10^–9^ μg / MB, equal to around 1.69 × 10^6^/MB; Fig. S4), which is indicated by the lower standard deviation. In addition, there is a tendency towards higher coupling efficacy. Overall, these findings indicate that aminolysis results in efficient and controllable coupling of cRGD to the surface of PBCA-MB, and furthermore preserves the MB integrity and US response.

### In vitro cRGD-MB binding to TNF-α stimulated HUVEC

**Fig. 3:**
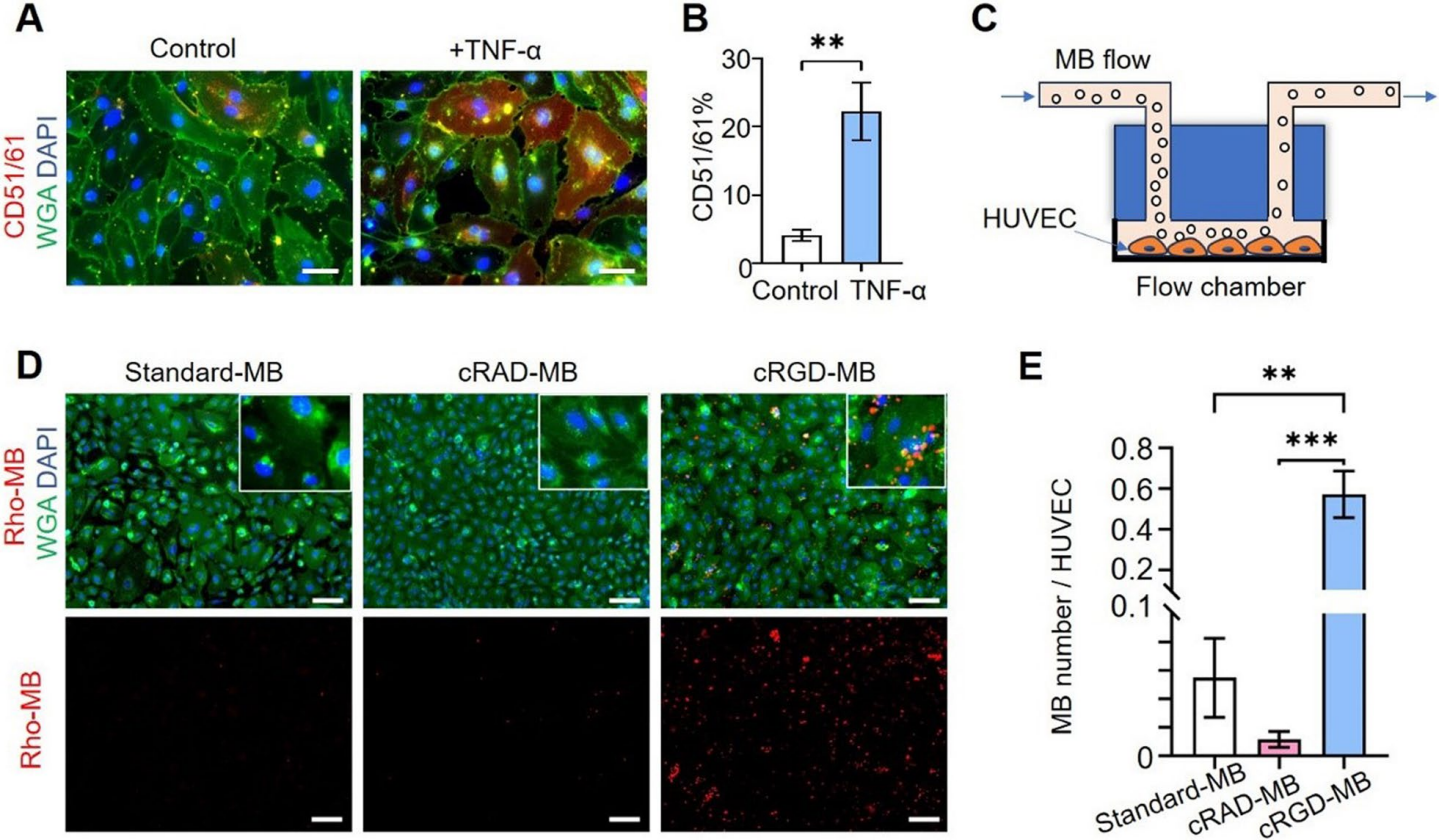
In vitro binding of cRGD-MB to HUVEC under flow conditions. **A** Fluorescence images of non-stimulated (control) or TNF-α stimulated HUVEC. The nuclei, cell membrane and α_v_β_3_ integrins were stained with DAPI (blue), wheat germ agglutinin-488 (WGA, green) and CD51/61 antibody (red), respectively. Scale bar = 50 μm. **B** Quantitative analysis indicating significantly higher area fraction of α_v_β_3_ integrin in HUVEC stimulated with TNF-α. Data are represented as mean ± SD. **C** Schematic showing the in vitro setup of the flow chamber. Petri dishes with cultured HUVEC were connected to a flow chamber, in turn perfused with an MB solution (standard-MB, cRAD-MB, or cRGD-MB) at 0.25 mL/min. **D** Representative fluorescent images of the three different, rhodamine-labeled MB types binding to TNF-α stimulated HUVEC. The nuclei, cell membrane and MB were labeled with DAPI (blue), WGA (green) and rhodamine (red), respectively. Scale bar: 100 μm. **E** Number of bound MB per cell. cRGD-MB displayed significantly higher binding to TNF-α stimulated HUVEC compared to both controls. Data represent mean ± SD of three independent MB batches. Statistical comparisons were performed using unpaired t-tests in panel B, while for panel E comparisons were performed using one-way ANOVA. **p < 0.01 and ***p < 0.001

In the next steps, we investigated the binding of cRGD-MB to inflamed cells under in vitro flow conditions. The experiments were performed using human umbilical vein endothelial cells (HUVEC), which overexpress α_v_β_3_ integrins under inflammatory conditions. To induce α_v_β_3_ integrin overexpression, HUVEC were exposed to tumor necrosis factor-alpha (TNF-α). The upregulation of α_v_β_3_ integrin was confirmed via immunofluorescence imaging, showing a 5-fold increase for HUVEC incubated with TNF-α compared to untreated HUVEC (p < 0.01; Fig. 3A–B).

Upon stimulating the HUVEC with TNF-α, we studied the specific binding of cRGD-MB labeled with Rhodamine under physiological conditions. A flow chamber system including TNF-α stimulated HUVEC was used to evaluate the ability of targeted MB binding in a dynamic environment alike the tumor microvasculature (Fig. 3C). As shown in Fig. 3D, E, the number of MB bound to HUVEC was more than 10-fold and 50-fold higher for cRGD-MB (0.57 ± 0.11 MB / cell) than for standard-MB (0.05 ± 0.02 MB / cell) and cRAD-MB (0.01 ± 0.002 MB / cell), respectively. These findings demonstrate that cRGD-MB bind efficiently to inflamed endothelial cells under flow conditions in vitro.

### Ex-vivo cRGD-MB binding to TNF-α stimulated mouse aortas

**Fig. 4:**
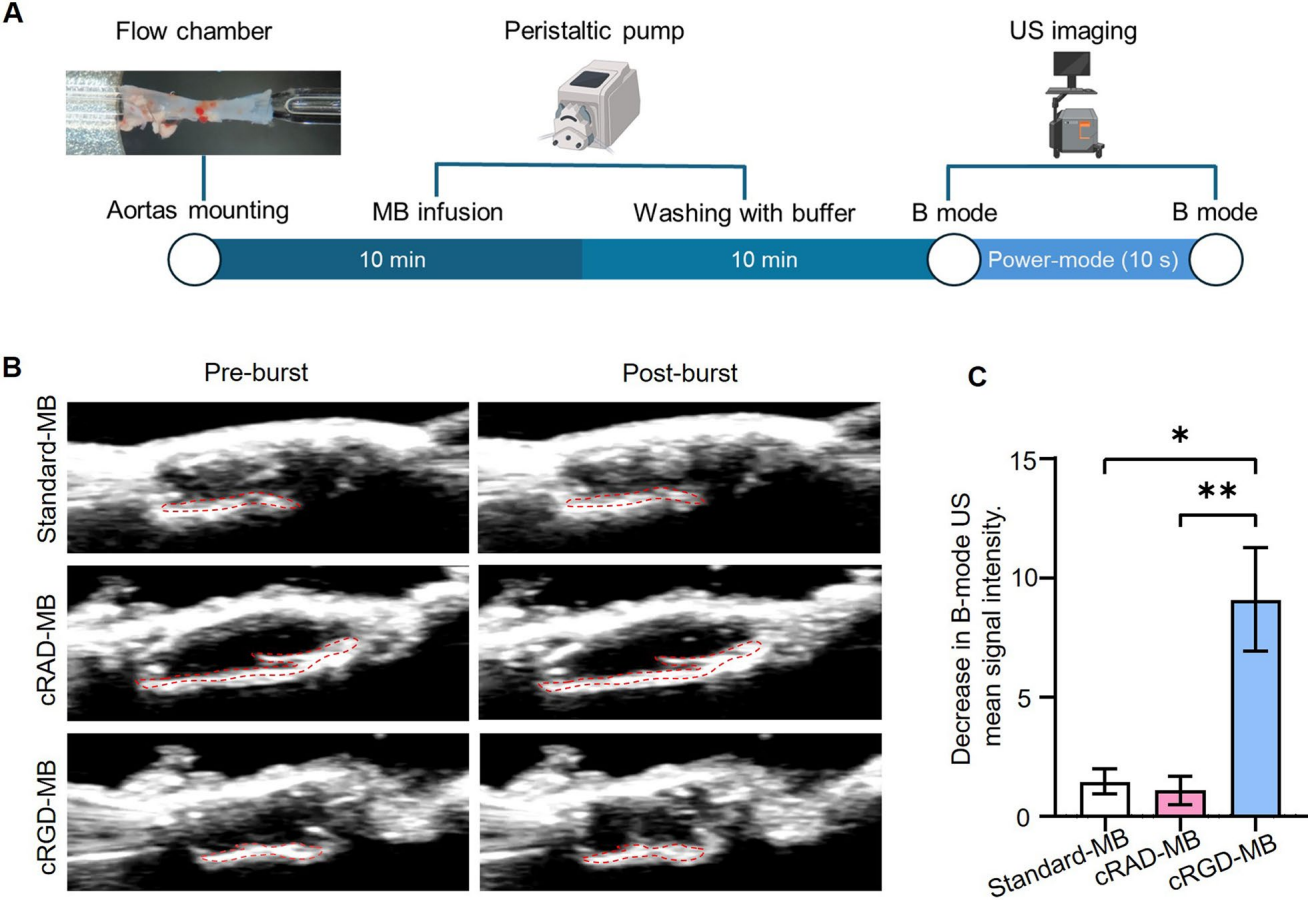
Ex vivo binding of cRGD-MB to TNF-α stimulated mouse aortas. **A** Experimental setup showing ex vivo mouse aortas mounted on a flow chamber. The aortas were perfused with an MB solution for 10 min, followed by washing steps to remove unbound MB. Finally, US was employed to quantify the number of bound MB by comparing the B-mode signal intensity before and after a destructive pulse. **B** Representative B-mode images before and after the destructive US pulse. High echogenicity was observed at the vessel wall following the infusion of cRGD-MB. Upon application of a destructive US pulse, B-mode US signals diminished within the highlighted area (Red). **C** Decrease in B-mode signal at the vessel wall before and after the destructive US pulse. For cRGD-MB there is a significant decrease in mean B-mode US signal intensity, indicating efficient binding to the TNF-α stimulated aortas. Data represent means ± SD of three independent MB batches. Statistical comparisons were performed using one-way ANOVA. *p < 0.05, **p < 0.01

Next, we investigated the binding of cRGD-MB under ex vivo conditions using mouse aortas. First, the mouse aortas were mounted on a flow chamber and stimulated with TNF-α (Fig. 4A). Next, an MB solution was infused and allowed to circulate for 10 min, followed by the removal of unbound MB. Finally, the bound MB were exposed to destructive US, and the number of bound MB was evaluated as the change in B-mode signal intensity before and after the destructive pulse. In contrast to standard-MB and cRAD-MB, we observed a strong signal change in B-mode US before and after the destructive US pulse for cRGD-MB (indicated by yellow dashed ROI; Fig. 4B). Quantitative analysis showed a more than 5-fold higher decrease in B-mode signal intensity compared to both standard-MB and cRAD-MB (p < 0.05; Fig. 4C).

### In vivo binding of cRGD-MB to 4T1 tumors

**Fig. 5:**
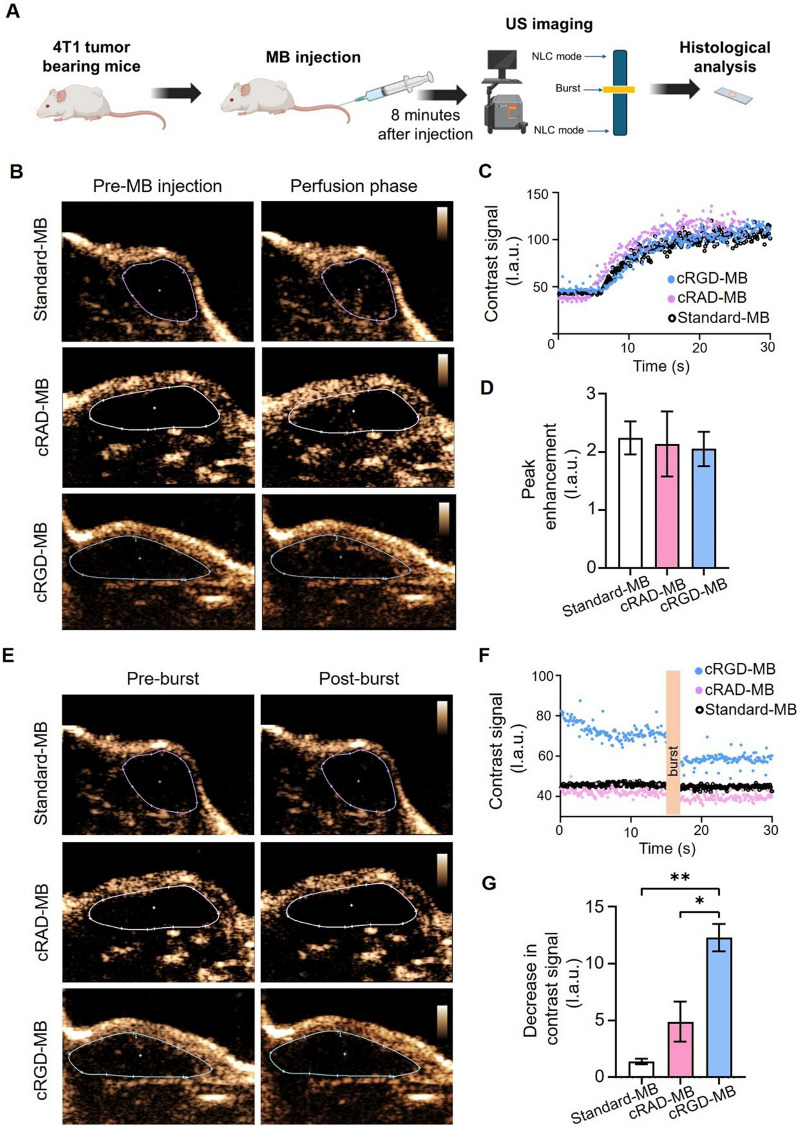
In vivo binding of cRGD-MB to α_v_β_3_ in mice tumors. **A** Experimental design of the in vivo experiments. MB were injected intravenously into 4T1 tumor-bearing mice for molecular US imaging using the destruction-replenishment method. Figure was created by Biorender.com. **B** Representative NLC-mode images of 4T1 tumors before and 30 s after MB injection (perfusion phase). **C** Representative contrast signal time-intensity curves confirming that the inflow of MB into the tumors is comparable for all formulations. **D** The peak enhancement of contrast signal in tumors demonstrated no significant differences between the groups, indicating that the different MB show a comparable contrast signal. **E** Representative NLC-mode images of tumors before and after the burst sequence. After 8 min of MB circulation, cRGD-MB had bound to their target and disintegrated after the application of a high-intensity burst sequence. **F** Representative contrast signal time-intensity curves indicating that after eight minutes more cRGD-MB had bound to the vessel wall compared to standard-MB and cRAD-MB, as cRGD-MB showed both a higher initial signal intensity and decrease in signal intensity after the burst. **G** Decrease in the US contrast signal intensity before and after applying the burst sequence. cRGD-MB (n = 5) showed the highest decrease in US contrast signal intensity than standard MB (n = 5) and cRAD-MB (n = 5). Data are represented as mean ± SD. Statistical comparisons were performed using one-way ANOVA. *p < 0.05, **p < 0.01

We finally evaluated whether cRGD-MB can be used to target the vasculature of 4T1 tumors in vivo, which are typically characterized by an overexpression of α_v_β_3_ integrin [25]. MB were intravenously injected and, after 8 min, US imaging was employed to study the MB binding to the vessels, followed by histological analysis to validate the integrin expression in tumor vessels (Fig. 5A). Upon MB administration, we first confirmed the inflow of MB in tumors (Fig. 5B–C) using US in NLC-mode. There was an early US signal peak in the large vessels, which then rapidly decreased (Fig. S5). The blood half-life of cRGD-MB was roughly estimated to be 5.7 s. An accurate measurement of the microbubble blood half-life is difficult due to different factors such as the dependence of the signal intensity on physical MB parameters, e.g. their size and in vivo stability. In this regard, MB that give a strong signal might be less stable and therefore eliminated earlier from the blood, whereas more stable MB with a longer circulation time generate less signal. As expected, we did not observe any significant differences in the peak enhancement between cRGD- and nonspecific (untargeted or cRAD-targeted) MB, indicating that they reached the tumors in comparable concentrations and have similar US inflow kinetics and acoustic properties in vivo (Fig. 5D). Eight minutes after MB injection, cRGD-MB were still visible in the tumor, while nearly no signal was observed for standard-MB and cRAD-MB. We quantified the number of bound MB using the destruction-replenishment technique, i.e., by quantifying the change in US signal before and after a destructive sequence. After the destructive pulse, the contrast signal generated by cRGD-MB decreased immediately, while hardly any change was observed for nonspecific MB groups (Fig. 5E–F). cRGD-MB displayed a more than 2–3 fold higher decrease in US contrast signal when compared to standard-MB and cRAD-MB (p < 0.05; Fig. 5G), respectively. Finally, after excising the tumors, we did not observe any significant differences in α_v_β_3_ expression between the MB groups (Fig. S6), underlining the hypothesis that the US signal loss for cRGD-MB can be attributed to its binding to the angiogenic vessels.

## Discussion

Antibody- or peptide-functionalized MB have been widely used in US molecular imaging [26–28] and have shown the potential to detect angiogenesis, inflammation, and thrombus formation [29–31] within preclinical setups. To this end, various intravascular targets have been proposed for MB targeting, such as integrins, selectins, and cell adhesion molecules [30, 32]. To develop targeted MB, polymer-based materials offer versatile strategies for functionalizing the MB shell with targeting ligands. In this regard, Li et al. employed polysaccharides act as nucleophiles to initiate the polymerization of butyl-cyanoacrylate (hydroxyl groups of the polysaccharide attack the C = C of monomer) to produce thrombus targeting polysaccharide-coated PBCA-MB [33]. However, this approach is specific to polysaccharide modifications. In the case of introducing peptides and proteins, only hydrolysis followed by carbodiimide chemistry were used to covalently couple peptides and proteins onto the MB surface. To date, PBCA-based polymeric MB have been functionalized with streptavidin or other peptides and antibodies recognizing α_v_β_3_ integrin, vascular endothelial growth factor receptor 2 (VEGFR2), P-and E-selectin, or vascular cell adhesion molecule 1 (VCAM-1) [16, 20, 31, 34, 35]. The efficiency of hydrolysis and carbodiimide chemistry is pH- and concentration-dependent, respectively [36]. Hydrolysis usually requires high pH conditions of ~ 10 to cleave the ester bonds into carboxyl groups on the MB surface. However, high pH conditions can lead to depolymerization of polymer chains, thus resulting in disruption of MB and loss of their acoustic property [23, 33]. Carbodiimide chemistry, in turn, generally gives optimal coupling at acidic conditions (pH 4.5–7.2) and, thus, the mismatch in pH conditions further increases the number of reaction steps [37, 38]. Consequently, coupling strategies that are simple, efficient, and less time-consuming would be advantageous for clinical translation of actively targeted PBCA-MB.

Here, we employed single-step aminolysis for surface functionalization of PBCA-MB. Aminolysis is typically a nucleophilic substitution reaction wherein a nucleophile replaces an electrophile [39]. Keeping this in mind, several factors were considered to implement aminolysis for conjugating cRGD on PBCA-MB. Firstly, cRGD peptides were chosen as a model ligand not only because it can strongly bind to α_v_β_3_ integrin, but also because it contains a highly active primary amine that can play the role of a nucleophile [40]. Secondly, the ester bond of the PBCA-MB acts as an electrophile. However, the ester bonds present on the PBCA polymer chain may show low reactivity [41, 42]. To enhance the reaction efficiency, we used lithium methoxide as a base catalyst for aminolysis. It was reported that the efficiency of ester cleavage increases in presence of lithium [43–46]. This is due to the coordination of the lithium cations to the ester carbonyl group. This results in chelates with increased electrophilicity, thus making the ester carbonyl group more prone to attack of amines. Thirdly, we performed the aminolysis reaction at pH 8 as a milder condition to protect MB from degradation while this reaction is optimal above pH 8 [47]. Overall, these conditions enabled the cRGD peptide to exhibit nucleophilic properties and cleave the ester bond of PBCA, which is then replaced with amide bonds and hydroxyl groups. Our findings showed that aminolysis successfully enabled the conjugation of cRGD on PBCA-MB, which is in line with other reports [48, 49]. Furthermore, aminolysis was shown to preserve the cRGD-MB concentration and their acoustic signal.

Actively targeted MB have been used for US molecular imaging of inflammatory and angiogenic tumor vessels [2, 50]. Under these pathophysiological conditions, vessels are typically characterized by an overexpression of VEGFR2, vascular cell adhesion molecules, selectins, and integrins [51]. For instance, BR55, a clinical grade VEGFR2-targeted MB, was developed to enable US molecular imaging of tumor angiogenesis [7]. Regarding vascular cell adhesion molecules, Curaj et al. illustrated that VCAM-1 targeted PBCA-MB could efficiently monitor vascular damage and endothelial recovery after arterial interventions [52]. Concerning selectins, Liu et al. demonstrated that E selectin-targeted PBCA-MB were able to actively bind to activated endothelium in vitro and enabled US molecular imaging of ex vivo activated carotid arteries [53]. In the case of integrins, MB coupled to cRGD by hydrolysis and carbodiimide were found to actively target the endothelium of 4T1 tumors, carotid arteries after injury, and tissue-engineered vascular grafts [35, 54, 55]. Although we only employed the aminolysis method to cRGD in this study, coupling of the other ligands is presumed to work as well as they also contain amine groups. Which of the ligands is the best for clinical translation, has still to be evaluated. In this context, however, the cost effectiveness of cRGD will also be a decision criterion.

Following up on these encouraging findings, there is room to further improve the performance of actively targeted PBCA-MB generated by aminolysis. In particular, the amount of cRGD peptides on the MB surface needs to be further optimized for clinical translation, such that it is sufficient for active targeting of endothelium without compromising the material costs for additional ligands and their elimination by the mononuclear phagocyte system. Furthermore, as the expression of integrins on tumor vessels is highly variable between different tumor types and might even vary among tumors of the same histological type, it is indicated to carefully check integrin expression on tumor vessels on either human pathological samples or by performing further experiments on different preclinical tumor models.

While aminolysis for synthesizing cRGD-MB provides a strong foundation for clinical translation, several critical considerations must be addressed before clinical studies can be started. Firstly, the synthesis process must be scaled up to meet GMP (Good Manufacturing Practice) standards. This requires appropriate methods and equipment for the synthesis, purification and stabilization of the MBs. In particular, during synthesis, it is crucial to use a suitable stirrer and reactor to ensure uniform mixing of a large volume without temperature gradient. Another challenge is the MBs’ floating, which complicates handling compared to other pharmaceuticals. To ensure good batch-to-batch consistency, it is essential to achieve even distributions of the MBs in the solution, particularly during the filling process.

To fulfill GMP conditions, product quality and intermediates must be verified throughout production, processing, packaging, and storage. Therefore, MBs will be lyophilized, to increase their shelf-life. Establishing sensitive and reproducible analytical methods is crucial for quality testing. Notably, the aminolysis has one fewer synthesis step than the hydrolysis protocol, simplifying production, documentation, and quality testing. Suitable sterilization methods must also be investigated to reduce bacterial load and minimize possible side effects. Finally, safety and toxicology tests have to be conducted in two different animal models. The resulting data will support the application for a clinical trial.

We believe that, in the future, our aminolysis procedure can not only be utilized for the modification of MB but also holds promise for the modification of other PBCA-based particles, such as nanobubbles or nanoparticles. Furthermore, the aminolysis procedure can be further leveraged to introduce amine-functionalized PEG linkers. This approach facilitates efficient bioconjugation of a wide variety of ligands through established techniques, e.g. click chemistry [56]. Moreover, our findings could also support the development of a PBCA-based theragnostic system in personalized medicine since PBCA-based MB and nanoparticles are excellent carriers for drug loading as well [57].

## Conclusion

We developed a novel aminolysis protocol for coupling targeting ligands on PBCA-MB. This coupling approach offers a simple strategy to prepare reproducible cRGD-MB formulations, establishing the groundwork for their translational use in US molecular imaging of pathophysiological conditions and beyond.

### Supplementary Information

Supplementary Material 1.

## Data Availability

No datasets were generated or analysed during the current study.
