# Supplementary material for: Aminolysis-mediated single-step surface functionalization of poly (butyl cyanoacrylate) microbubbles for ultrasound molecular imaging
Source: J Nanobiotechnology. 2024 Sep 1;22:528. doi: 10.1186/s12951-024-02806-9 (PMC11367926; doi:10.1186/s12951-024-02806-9)
Supplement: Supplementary file 1 — Supplementary Material 1. [file 12951_2024_2806_MOESM1_ESM.docx]

**Aminolysis-Mediated Single-Step Surface Functionalization of**

**Poly(butyl cyanoacrylate) Microbubbles for Ultrasound Molecular Imaging**

Junlin Chen, Bi Wang, Anshuman Dasgupta, Celine Porte, Lisa Eckardt, Jinwei Qi, Marek Weiler, Twan Lammers, Anne Rix, Yang Shi, Fabian Kiessling*

Institute for Experimental Molecular Imaging, RWTH Aachen University, Aachen 52074, Germany

Corresponding author: [fkiessling@ukaachen.de](mailto:fkiessling@ukaachen.de)

**Immunoprecipitation with magnetic beads**

To assess the targeting efficiency of microbubbles (MB) using the Dynabeads™ Protein G Immunoprecipitation Kit (Thermo Fisher, MA, USA), procedures were performed following the manufacturer’s protocol. Briefly, magnetic beads were first coated with a monoclonal CD51/CD61 antibody (0.05 mg/mL; Thermo Fisher, MA, USA) for 30 minutes. The beads were then washed with the antibody binding and washing buffer provided in the kit. Subsequently, 200 μL of MB suspension (5 × 10^7^/mL) was added to the beads and incubated for 30 minutes to allow binding. Following incubation, the MB-bound beads were collected using a magnetic separator, and the unbound MBs were transferred to another tube for concentration measurement using a Coulter counter. The targeting efficiency (%) was calculated by dividing the change in MB concentration before and after immunoprecipitation by the MB concentration before immunoprecipitation.

**Colorimetric peptide assay**

To quantify the ligand density per MB, the micro BCA protein assay kit (Thermo Fisher, MA, USA) was used. Working reagents were prepared according to the manufacturer's protocol. 50 μL MB (5 ×10^8^ /mL) were diluted in 450 μL acetonitrile (ACN) / water (1:1). 150 μL of the diluted samples were added to 96-well plates and mixed with 150 μL of the working reagent. The mixtures were incubated at 37 °C for 2 hour and their absorbances were measured at 562 nm using a plate reader.


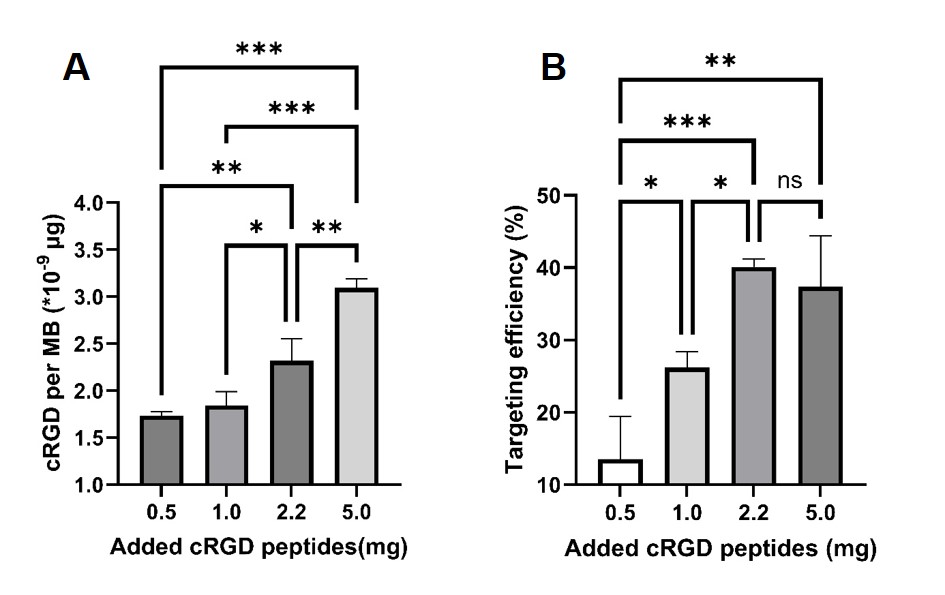


**Fig.S1** **Optimization of the cRGD dosage for the aminolysis based conjugation.** A) The cRGD peptide load per MB increases with the amount of added cRGD during aminolysis. B) Targeting efficiency was not further increased when adding more than 2.2 mg cRGD during aminolysis. *p<0.05, **p<0.01, ***p<0.001.


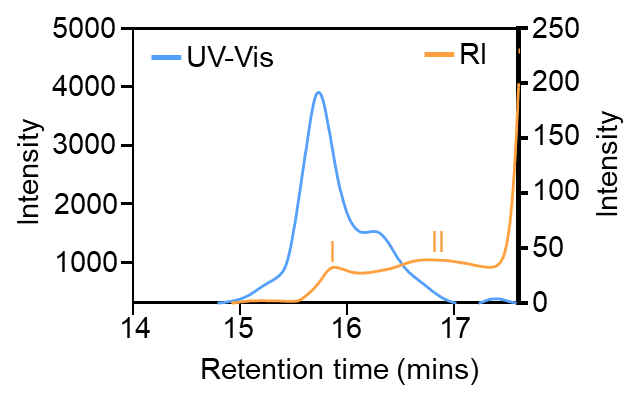


**Fig. S2** The GPC diagram of Cy3-MB under UV-Vis and RI detectors.


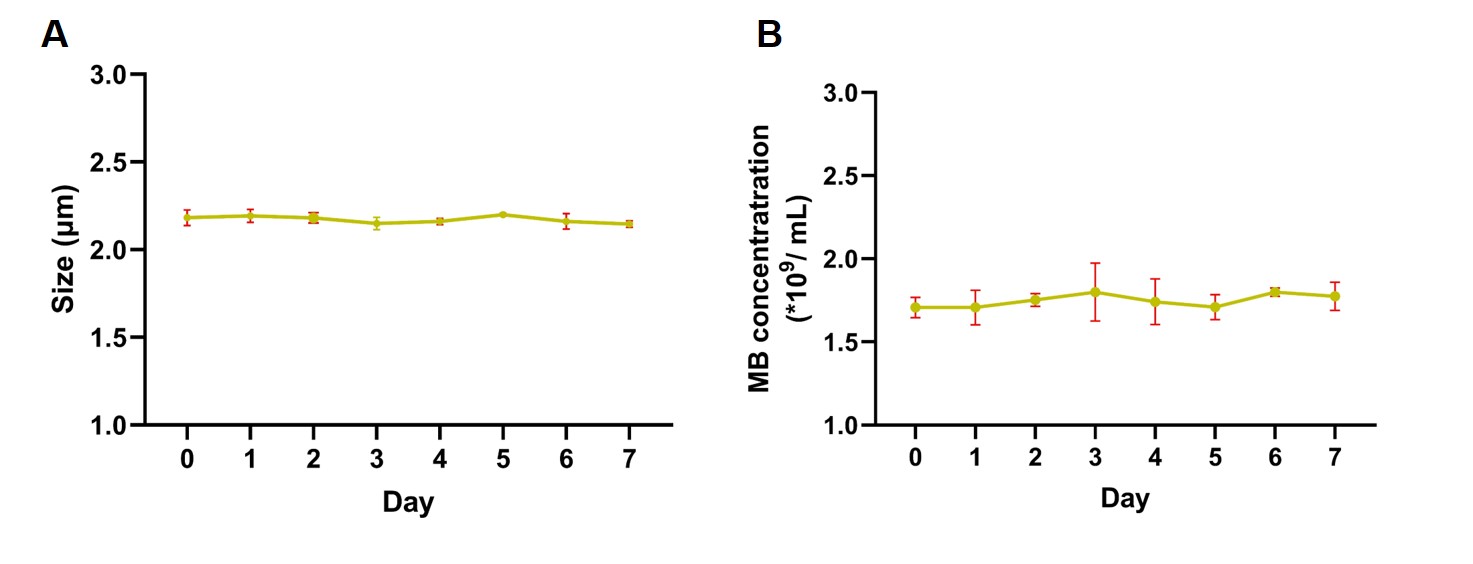


**Fig.S3** MB size(A) and number (B) do not change in suspension over 7 days.


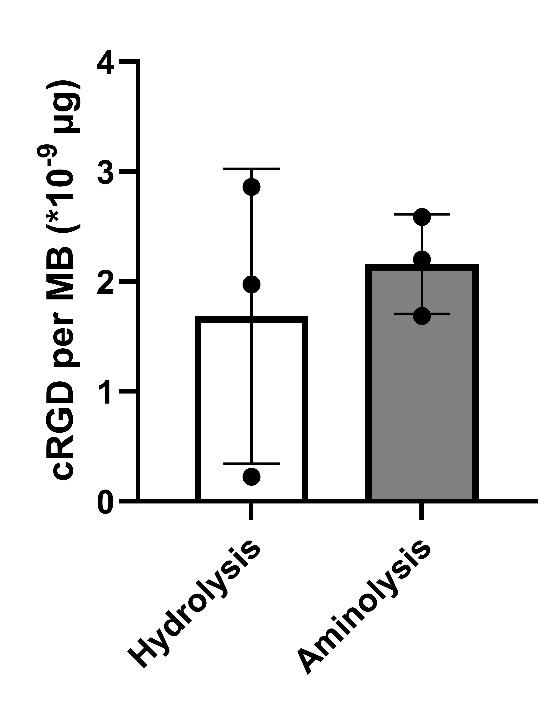


**Fig. S4** The Pierce^TM^ BCA colorimetric peptide assay indicates that aminolysis has less batch-to-batch variation than hydrolysis and tends to result in a higher conjugation efficacy.


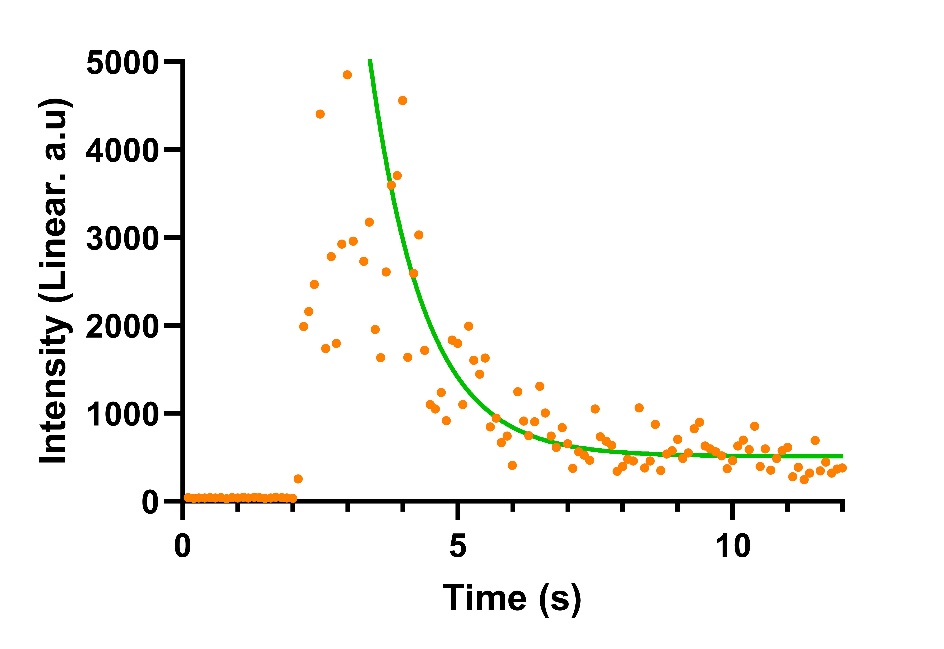


**Fig. S5** The ultrasound time-intensity profile in a large vessel after injection of the cRGD-MB.


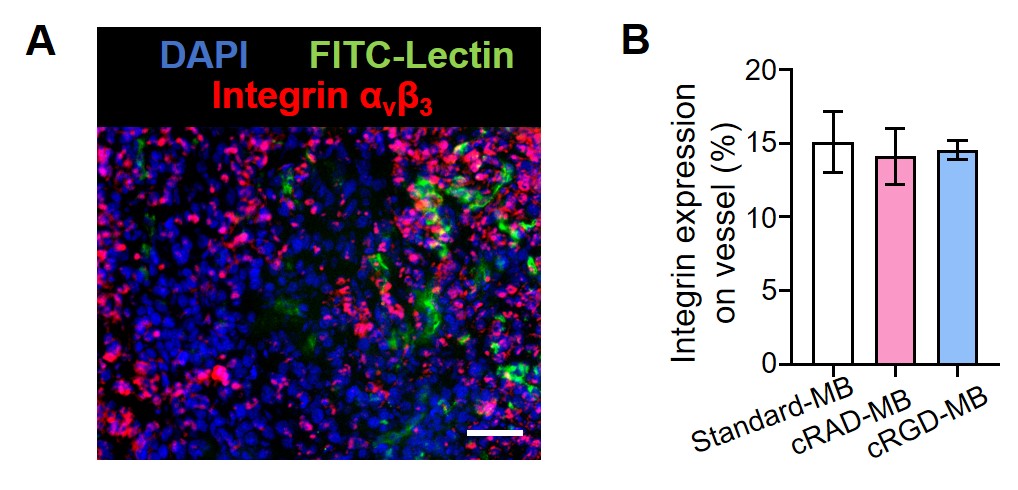


**Fig. S6** **α_v_β_3_ integrin expression in mouse breast tumor.** **A** Representative fluorescence image of α_v_β_3_ integrin (red) within a mouse breast tumor. FITC-lectin (green) was used to label the perfused blood vessels before sacrificing the mice. α_v_β_3_ integrin expression is observed on vessels but also on perfused tumor cells. Scale bar =100 μm. **B** Quantification of α_v_β_3_ integrin expression on perfused vessels. No significant differences in α_v_β_3_ integrin expression were found between groups.
